# Prediction of Mortality Using On-Line, Self-Reported Health Data: Empirical Test of the Realage Score

**DOI:** 10.1371/journal.pone.0086385

**Published:** 2014-01-17

**Authors:** William R. Hobbs, James H. Fowler

**Affiliations:** 1 Division of Social Sciences, University of California San Diego, La Jolla, California, United States of America; 2 Division of Social Sciences, Division of Medical Genetics, University of California San Diego, La Jolla, California, United States of America; Cardiff University, United Kingdom

## Abstract

**Objective:**

We validate an online, personalized mortality risk measure called “RealAge” assigned to 30 million individuals over the past 10 years.

**Methods:**

188,698 RealAge survey respondents were linked to California Department of Public Health death records using a one-way cryptographic hash of first name, last name, and date of birth. 1,046 were identified as deceased. We used Cox proportional hazards models and receiver operating characteristic (ROC) curves to estimate the relative scales and predictive accuracies of chronological age, the RealAge score, and the Framingham ATP-III score for hard coronary heart disease (HCHD) in this data. To address concerns about selection and to examine possible heterogeneity, we compared the results by time to death at registration, underlying cause of death, and relative health among users.

**Results:**

The RealAge score is accurately scaled (hazard ratios: age 1.076; RealAge-age 1.084) and more accurate than chronological age (age c-statistic: 0.748; RealAge c-statistic: 0.847) in predicting mortality from hard coronary heart disease following survey completion. The score is more accurate than the Framingham ATP-III score for hard coronary heart disease (c-statistic: 0.814), perhaps because self-reported cholesterol levels are relatively uninformative in the RealAge user sample. RealAge predicts deaths from malignant neoplasms, heart disease, and external causes. The score does not predict malignant neoplasm deaths when restricted to users with no smoking history, no prior cancer diagnosis, and no indicated health interest in cancer (p-value 0.820).

**Conclusion:**

The RealAge score is a valid measure of mortality risk in its user population.

## Introduction

Between 70 and 90 percent of Americans seek out health information online [1], [2]. Personalized, automated health risk assessments are an especially prominent source of information and may be useful because they aggregate many different sources of health information [3]–[6]. In particular, these evaluations may have a positive impact on health outcomes since tailored health information is thought to be more effective than general health information in bringing about changes in health behaviors [7].

However, there is also widespread concern that online health information may be of low quality and therefore its use might mislead patients in their self-management [8], [9]. Many influential studies suggest that healthcare professionals should be careful only to recommend high quality or credible online sources of health information (see, for example, [3], [10], [11]). Therefore, it is important to validate these online sources if they are to be used by patients, healthcare providers, and the broader public.

Here, we evaluate a personalized health measure called “RealAge” that has been assigned to over 30 million individuals via the internet (see www.realage.com) in the past 10 years. The premise of the RealAge score is that a person in very good health has a “real” biological age that is younger than his or her chronological age. For example, a 35 year old with a RealAge of 30 is someone whose health and potential longevity are more like those of average 30 year olds than average 35 year olds. Conversely, a person in poor health may have a RealAge that is higher than their chronological age. We therefore expect the difference between the RealAge score and chronological age (the “RealAge delta”) to be predictive of mortality, with negative values indicating reduced risk and positive numbers indicating increased risk. Moreover, if the score is scaled correctly, then the increased risk resulting from an extra unit in the RealAge score should be of the same magnitude as the increased risk due to an extra year of life.

Future research should further evaluate the contributions of specific inputs to online health risk assessments (from self-reports, in particular), measure their accuracy in broader populations, and test the efficacy of health advice provided to survey-takers.

## Data and Methods

This research was approved by the Institutional Review Board at the University of California, San Diego under protocol 111781, “RealAge Collaboration”, and by the Committee for the Protection of Human Subjects at the California Department of Public Health under protocol 12-04-0053, “RealAge Collaboration - Prediction of Mortality Risk Using Online Health and Health Interest Data”.

### Data

To test the RealAge score, two data sources that cover the time period 2000 through 2010 were linked: online surveys of 188,698 RealAge users with complete identifiers (first name, last name, and date of birth) living in California and the California Death Statistical Master File. The RealAge online surveys contain extensive information on user demographics, health status (including health conditions such as diabetes and cancer), health behaviors, and family health history (see www.realage.com for the current implementation of the survey). While we do not possess detailed information on the RealAge prediction procedure, we note that reports by the company indicate that important contributors to RealAge scores in its user base are: high blood pressure and cholesterol, marriage, health insurance, employment status, alcohol and tobacco consumption, and diabetes management.

In addition to the information collected from users to construct their personal RealAge scores, the survey also requests information on health interests, caregiver responsibilities, and a myriad of conditions and health behaviors with relatively unknown relationships to mortality risk (relationships less well-established than high blood pressure or diabetes, for example). Users are allowed to take the survey more than once, but we restrict our analyses to the last survey taken prior to 2011 (corresponding to the end of our observation period).

The California Death Statistical Master File (CDSMF) is the most complete record of deaths in California. It is maintained and made available to researchers by the California Department of Public Health. The file includes both resident and non-resident deaths, and contains information such as first and last name, date of birth, date of death, and underlying cause of death. The RealAge data (188,698 users) was linked to this database using a one-way, cryptographic hash of first name, last name, and date of birth. Identifiers (duplicate first name, last name, and date of birth records) that occur more than once in either dataset were excluded from the final analysis (these accounted for 2% of the data). Analysis data contained only a non-personally-identifiable record number for each user.

Records with dates of birth for January 1 of each year and the first of each month were excluded from the full-identifier sample since histograms show an excess of birthdays on these dates, suggesting users did not take time to fully enter their birth date (these account for 4% of the data). Users for whom RealAge maintained conflicting residences (e.g. more than one state of residence or conflicting zip code/state of residence) were also removed (these account for 7% of the data). 3% of users do not list a gender and these observations were also removed. This process leaves the 188,698 records noted above. We grouped causes of death into three categories (malignant neoplasms/cancers, heart diseases, and external causes excluding same-level falls) using designations from the *International Classification of Diseases, Tenth Revision* [12].

### Sample

The vast majority of RealAge respondents in our data completed the survey in years 2005 through 2009. Therefore, our analysis is most relevant for evaluating whether the RealAge score is useful for predicting mortality risk in the short-term (one to five years). Our results are more generalizable if we assume that respondents have not recently altered their health behaviors (i.e. their current responses are representative of their health behaviors and conditions for a long period preceding the survey).

The RealAge score is most relevant to the population actually using the metric. Table 1 describes the demographic characteristics of the RealAge California user population with complete identifiers. The sample is predominantly female and white, most users are married, and the average user possesses a college education or higher.

**Table 1:** Demographics in RealAge California Full Identifier Sample.

Age	Mean: 48
	SD: 13
Female	73%
White	73%
Married	63%
College-educated (or higher)	49%
Ex-Smoker	33%
Current Smoker	14%

It is also important to consider whether RealAge users are representative in terms of causes of death and death rates compared to the US population. Although the RealAge sample is more middle-aged, on average, Figure S1 in Appendix S1 shows that death rates in the RealAge population are comparable to those in the US population as a whole up to about age 60, and after that tend to be somewhat lower. Figures S2 and S3 in Appendix S1 show the distribution of the RealAge delta in the California sample and in the full US RealAge population, respectively. The distribution of the score does not substantively differ between the two groups. Table 2 shows the count of different causes of death in our sample, along with average follow-up times by cause of death. Tables S1, S2, and S3 in Appendix S1 show causes of death by age group. Figures S4, S5, and S6 in Appendix S1 show the distribution of the RealAge delta by age group among deceased and surviving users in-sample.

**Table 2:** Causes of Death in RealAge California Full Identifier Sample.

Cause of Death	Count	Follow-Up* Mean	Follow-Up Median
Cancer	339	1114.6	917
– Lung Cancer	94	1090.8	899.5
– Breast Cancer	34	958.8	831
Heart Disease	252	1022.3	883
External Causes	104	937.3	785
– Unintentional Injury	61	960.1	842
– Suicide	32	863	730
Chronic Lower Respiratory	59	1020.3	887
# of users in sample	188,698		
# deceased in sample	1,046		
* Days from date of registration to date of death.			

### Model Description and Use for Scale Comparison

We use a series of Cox proportional hazards models ( [13], a standard survival analysis tool [14]) to evaluate the scale and accuracy of the RealAge score in predicting mortality within its user population. For the scale evaluation, we are interested in comparing the model coefficients for the RealAge score to the coefficients for chronological age, so we test three survival models, one with chronological age, one with RealAge, and one that includes both chronological age and the “RealAge delta”, which is simply the RealAge minus chronological age. Since RealAge is itself a function of age and other factors, the combined model allows us to test 1) whether RealAge score is contributing to predictive power of the model over and above the simple relationship between age and mortality; 2) whether the model is scaled appropriately.

The Cox proportional hazard model uses duration of survival to estimate the ratio of the predicted risk of death to a baseline risk of death that can vary arbitrarily with time. In our case, duration is the time since the user last took the RealAge survey and this value is censored if the user has not yet died on the last day of our time range of observations (December 31, 2010). Also, each of the models estimate here is stratified by gender, however, we do not report the results separately because they do not substantively differ (likely because the models examined here have already been re-scaled to account for gender differences).

### Assessment of Model Performance

In addition to assessing the significance and scale of the RealAge score, we also evaluate its ability to discriminate between relatively healthy and unhealthy respondents (see [15] for a comprehensive discussion of each of the assessment methods presented here). We first use the receiver operating characteristic curve (ROC curve) to test this discriminative ability. The ROC curve plots on the y-axis the sensitivity, or true positive rate, and on the x-axis one minus the specificity, or the false positive rate, across all cutoffs for the probability of death during follow-up. We further report Harrell's c (*c*-statistic) [16], which is equal to the area under the ROC curve for the binary deceased-not deceased outcome evaluated here, and is the probability that between two randomly chosen individuals, one deceased and one not, the decedent in the pair was assigned the higher mortality risk.

Further, we assess the goodness-of-fit between the predicted mortality risk and the observed mortality during follow-up. To do this, we plot observed mortality rates against those predicted by the model (a calibration plot), and draw a loess-smoothed line through these points. If the models are well-calibrated, this line should follow approximately the 45 degree line in the figure, or a perfect correspondence between predicted and observed mortality.

Because discrimination and goodness-of-fit do not necessarily convey sufficient information on changes in risk predictions for individual respondents, we also test the extent to which survey-takers would be given higher or lower risk predictions that better correspond to observed outcomes in an expanded model (e.g. RealAge) over the original model (Framingham ATP-III). The net reclassification improvement (NRI) [17] is the sum of 1) the proportion of deceased respondents assigned a higher mortality risk in the second model minus the proportion assigned a lower mortality risk; and 2) the inverse for surviving respondents, or the proportion assigned a lower mortality risk in the second model minus the proportion assigned a higher mortality risk. The integrated discrimination improvement (IDI) [17], also reported in this paper, is the NRI over all mortality risk cutoffs.

### Comparison to ATP-III

In addition to validating the scale and accuracy of the RealAge metric, it is also important to use the same data to test the RealAge accuracy against a model that includes traditional risk factors. For this test, we focus on the Framingham Adult Treatment Panel III (ATP-III), a model that predicts hard coronary heart disease (HCHD, which includes heart attack or coronary death) using the risk factors age, hypertension, smoking, diabetes mellitus, and total and high-density lipoprotein (HDL) cholesterol among individuals without diabetes or coronary heart disease. ATP-III is a less accurate predictor of HCHD than other metrics, including the Reynolds Risk Score [18], however, we are unable to evaluate the RealAge score against these stronger metrics. Only around 1% of users in our sample responded to questions on c-reactive protein and *family* coronary heart disease history. In particular, family coronary heart disease history was not included on the RealAge survey prior to 2010.

It is important to note that *both* the ATP-III and the RealAge score (likely based on information the ATP-III) are intended for use in cases with 10 years or more of follow-up. This validation is limited to only relatively short-term outcomes (with around 5 years of follow-up), and should later be extended to 10 or more years of follow-up.

There are two potential implementations of the ATP-III model described in 2001 ( [19]) and on the Framingham Heart Study website *(* http://www.framinghamheartstudy.org/risk/hrdcoronary.html *)*. The Framingham risk points model is a simple implementation of the risk factor categories that can be used by individuals and primary care physicians. A Cox proportional hazards model implementation of the risk factor prediction is an alternative, and perhaps more accurate implementation, but is difficult to carry out in the general population. Here, we implement the risk factor category model because it can be self-administered. Consistent with its intended use, we remove from all models individuals with diabetes (8% of the sample) and conditions of the circulatory system (angina, anemia, atrial fibrillation, heart attack, heart murmur/rheumatic fever/damaged heart valve, irregular heartbeat, mitral valve prolapse, peripheral arterial disease, and stroke) (8% of the sample). However, leaving these individuals in the models does not alter our results. We also limit the age range to 30 to 79 for consistency with the Framingham model.

The RealAge survey does not require that respondents know their exact blood pressure or cholesterol levels. Users who indicate that they do not know these numbers are asked to guess whether their numbers are low, average or high, but are not required to guess these numbers. To avoid bias from excluding these users from the model evaluation, we impute expected blood pressure and cholesterol levels using the covarying distributions of other variables (such as BMI). Table 3 shows the summary statistics of this sample before and after imputation.

**Table 3:** Summary Statistics in RealAge California Full Identifier Sample, ages 30 to 79, no diabetes or pre-existing circulatory conditions.

	Women-original	Women-imputed
Characteristics	n = 101,911	
Age, mean (SD)	49.2 (11.0)	
Total cholesterol, mean (SD) (%NA)	183.8 46.1 (65.0)	
- % low/medium/high (NA)	18.9/24.0/4.6 (52.5)	38.0/54.6/7.4 (0)
HDL cholesterol mean (SD)	62.8 18.3 (78.8)	
- % low/medium/high (NA)	2.6/24.4/15.5 (57.4)	3.1/64.1/32.8 (0)
Systolic Blood Pressure,mean (SD)	116.5 (140.5)	
- % low/medium/high (NA)	18.4/54.0/7.3 (20.3)	19.6/71.4/9.0 (0)
BP treatment, n (%)	11,021 (10.8)	
Current smoker, n (%)	11,107 (10.9)	
Heart disease deaths, n (%)	58 (0.06)	
	**Men-original**	**Men-imputed**
**Characteristics**	**n = 38,671**	
Age, mean (SD)	51.8 (11.9)	
Total cholesterol, mean (SD) (%NA)	172.3 (46.6) (57.9)	
- % low/medium/high (NA)	17.6/28.7/5.7 (48.0)	32.7/58.4/8.9 (0)
HDL cholesterol mean (SD)	53.6 17.9 (73.4)	
- % low/medium/high (NA)	3.3/30.2/13.5 (53.0)	3.9/69.5/26.6 (0)
Systolic Blood Pressure,mean (SD)	120.8 14 (47.8)	
- % low/medium/high (NA)	11.1/58.0/11.1 (19.8)	12.2/74.6/13.2 (0)
BP treatment, n (%)	5,427 (14.0)	
Current Smoker, n (%)	4,595 (11.9)	
Heart Disease Deaths, n (%)	77 (0.2)	

To complete this imputation for “guessing” users, we assign “low” responses to the lowest Framingham risk category, “average” to the second, and “high” to the third category (the 1st, 2nd, and 3rd out of 5 for total cholesterol and blood pressure, and the 1st, 2nd, and 3rd out of 4 for HDL cholesterol–this very slightly outperforms other choices, such as assignment to the 1st, 2nd, and 4th risk categories). We then impute exact levels using the mean of five bootstrapped estimates from covarying distributions of other self-reports and replace assignments when these estimates suggest that a user's high blood pressure or cholesterol levels would be in the 4th or 5th risk categories (this very slightly outperforms assignment to the 1st, 2nd, and 3rd risk categories alone). Assignment to the third highest, second highest, or highest categories does not substantively alter results (all Framingham models maintain a *c*-statistic, a measure of discriminative ability described above, between 0.807 and 0.814).

Further, because only around one-half of users in our sample report or attempt to guess their blood pressure (90.3% report or guess), total cholesterol (67.5% report or guess), and HDL cholesterol levels (56.5% report or guess), we impute unguessed (missing) data using low/average/high values using the median of five bootstrap estimates. These bootstrap estimates are again based on covarying distributions of other self-reports, including a high blood pressure indicator and body mass index. As noted above, the imputation reduces bias that could be introduced by removing these missing observations and ensures comparability to the full RealAge population. However, our comparisons to a complete-case only analysis suggests that this imputation for non-guessing respondents does not meaningfully alter our model results.

### Evaluation of Selection Concerns and Subgroup Differences

Individuals visit *realage.com* to obtain personal health information, and this selection into the RealAge sample potentially biases or alters the interpretation of our model estimates.

For example, cancer patients and survivors play an active role in obtaining health information about their condition [20], [21]. Relatively “unhealthy” (by the RealAge score) individuals might only seek out health information when facing serious illness, and a possible correspondence between the RealAge score and health-information seeking rather than health itself would inflate the estimates presented here. This hypothesis is in line with recent work arguing that internet users who search for health information broadly segregate into wellness-only and illness-only information-seeking groups [22].

Similarly, patients with terminal illness may seek out health information that directly corresponds to their doctors' prognosis, and the relative accuracy of this prognosis rather than personal health status (as indicated by the RealAge score) would affect the size of the model coefficients.

To address these concerns, we consider the possibility that among those users who die during the observation period, the RealAge score and the registration date of a RealAge user may be related to diagnosis/prognosis and time to death. To test this, we compare model results of users who registered at least two years prior to death to the full sample. If the results are insensitive to this confounder, then we expect RealAge to be equally predictive in this group.

Second, we consider the possibility that the RealAge score predicts only deaths among users with known and reported serious illness, such as cancer, but not among relatively healthy users. To evaluate this, we restrict our analysis to those users who have no cancer diagnosis and no expressed interest in cancer, no heart attack or stroke diagnosis and no interest in heart disease, and no indicated depression diagnosis and no interest in factors that lead to deaths from external causes. To further assess the possible health information contribution of the RealAge score, we restrict our analysis of cancer and heart disease deaths to users with no smoking history (we do not restrict to the external causes sample to no smoking, no depression diagnosis/interest because around 90% of users who die from external causes indicate smoking behavior and/or depression).

It is important to also test the validity of the RealAge score in specific sub-populations and by cause of death. For example, users under 45 are both much less likely to die than older users, and tend to die from very different underlying causes. To assess whether the RealAge score performs equally well for all ages, we run survival models for age groups 25 to 44, 45 to 64, and 65 to 84.

Last, we test the RealAge score using age in the base hazard. This model specification may be more appropriate for this analysis because it allows us to estimate the effects of age semi-parametrically [23], [24].

## Results

### Comparison of Age and RealAge Coefficients

Table 4 shows that the RealAge score predicts mortality on scale with age-based risk in the user population. Table S4 in Appendix S1 shows the log-likelihood for this table. A hazard ratio (HR) of 1.076 for age means that an individual's mortality risk increases by 7.6% over the baseline hazard at a given time following survey completion for each additional year of age. Identically, each additional year of RealAge also increases risk by 7.9%. When we decompose these two factors in the combined model, the estimates change only slightly – each year of age increases risk by 7.6% while each unit increase in the RealAge delta increases risk by 8.4%.

**Table 4:** Comparison of Age and RealAge.

	HR	(2.5%, 97.5%)	p
Age	1.076	(1.071, 1.081)	0.000
	HR	(2.5%, 97.5%)	p
RealAge	1.079	(1.075, 1.083)	0.000
	HR	(2.5%, 97.5%)	p
Age	1.076	(1.070, 1.081)	0.000
RealAgeDelta	1.084	(1.077, 1.090)	0.000
# of users in sample	188,698		
# deceased in sample	1,046		

Since the combined model controls for age, the significant coefficient on the RealAge delta suggests that the other health data incorporated into the RealAge is improving the predictive power of the model, and the approximately equal coefficients suggest that users have a risk of mortality that is “as if” they had a chronological age equal to their RealAge score. For example, a 35 year-old with a RealAge score of 30 has a RealAge delta of −5 and therefore a risk ratio over baseline hazard of 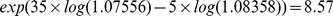. This is close to the risk ratio implied by the model with age alone for the average 30 year old: 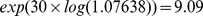.

### Comparison to Framingham Risk Scores

The results of the ATP-III risk factor model are presented in Table 5. The ATP-III model underperforms what we would expect in a population with accurate blood pressure and cholesterol levels. Because the point inputs in this model have been rescaled in order to be combined for an overall risk indicator, each of the model coefficients in Table 5 would be equivalent or nearly equivalent if they contributed as equally strong risk predictors. In particular, the coefficient on total cholesterol level suggests that user-reported cholesterol levels are uninformative. The model predicts coronary death primarily from age and current smoking behavior.

**Table 5:** Framingham Hard Coronary Heart Disease 10-Year Risk Model results.

	HR	(2.5%, 97.5%)	p
Age (points)	1.208	(1.151, 1.269)	0.000
Age:Current Smoker (points)	1.344	(1.220, 1.480)	0.000
Blood Pressure (points)	1.274	(1.069, 1.519)	0.007
Cholesterol (points)	0.894	(0.781, 1.023)	0.105
HDL Cholesterol (points)	1.199	(0.960, 1.496)	0.110
# of users in sample	140,582		
# deceased in sample	135		

**Figure 1:**
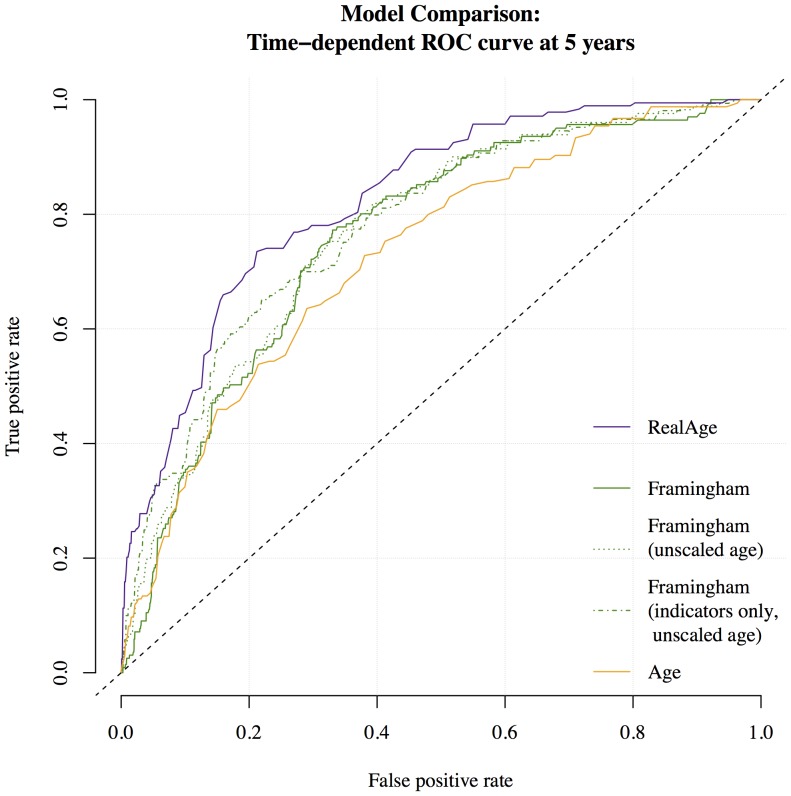
Comparison of RealAge, Age, and Framingham ATP-III models. (Time-dependent ROC curve at 5 years). This figure shows the discriminative ability of Cox proportional hazards models for age, age and RealAge, and the Framingham ATP-III scores. Models with ROC curves higher and to the left show better discrimination between unhealthy and healthy respondents–here meaning that, between a pair of randomly chosen respondents, the deceased user was assigned a higher predicted mortality risk. Calibration plots are included in Appendix S1 (Figures S7 through S11), and show that models with unscaled age are well-calibrated. Results in the text (the net reclassification improvement and the integrated discrimination improvement) show that the RealAge score provided improved case-by-case risk predictions compared to the Framingham models.

In Figure 1 we compare the accuracy of the RealAge and Framingham models using a receiver-operator curve that tests the accuracy of each model in predicting death for all possible model thresholds. As noted in the methods section, better fitting models yield fewer false positives and more true positives and therefore have curves that are higher and to the left. Further, the area under the curve (the *c*-statistic) corresponds to the probability that between two randomly chosen individuals, one who has died and one who has not, the mortality risk model assigned a higher risk of death to the decedent. Notice that the RealAge model performs best with a *c*-statistic of 0.847, while the age-only model yields a *c*-statistic of 0.784.

In comparison, the Framingham ATP-III model yields a *c*-statistic of 0.810. An alternative version of the Framingham model that uses unscaled age rather log-scaled age performs nearly identically with a *c*-statistic of 0.810. To assess whether simpler indicators of health upon which the ATP-III is based might yield a better model, we tried a third version in which we only used the most basic predictors of heart health: unscaled age, current smoker, an indicator for high blood pressure, and an indicator for high cholesterol. This version yielded a *c*-statistic of 0.807. Further, the integrated discrimination improvement (IDI) comparing the unscaled points model to the simplified, indicator-only model was not significant (0.000, 95% *CI*: [–0.001, 0.001], *p-value* = 0.698), supporting the hypothesis that specific, user-reported blood pressure and cholesterol levels do not contribute to improved risk predictions in this data. In contrast, the IDI for the RealAge model compared to the Framingham unscaled points model was highly significant (0.004, 95% *CI*: [0.002, 0.011], *p-value* <0.001). We note that we obtain substantively equivalent estimates from the net reclassification improvement (Framingham comparison: –0.096, 95% *CI*: [–0.186, 0.187], *p-value* = 0.831; RealAge to Framingham comparision: 0.314, 95% *CI*: [0.172, 0.433], *p-value* <0.001).

Tables S5 and S6 in Appendix S1 show the coefficient estimates for the unscaled age Framingham model and the simple risk factor-only (unscaled age) models, respectively. We also show in Appendix S1 Figures S7 through S11 the results of the calibration analysis. These figures show that all models except for the re-scaled, original version of the Framingham ATP-III are well-calibrated.

### Estimates by Age and Cause of Death

In Table S7, Table S9, and Table S11 in Appendix S1, we show that the RealAge score is predictive of death within each age group, including 25 to 44 (risk ratio for a unit change in age: 1.049, RealAge delta: 1.087), 45 to 64 (age: 1.064, RealAge delta: 1.094), and 65 to 85 (age: 1.086, RealAge delta: 1.070). Tables S8, S10, and S12 in Appendix S1 show substantively equivalent results with age in the base hazard. The RealAge score is more predictive among younger users than users 65 or older. The performance of the RealAge score under age 45 is notable because most deaths at these ages are from unintentional injuries, suicides, and murders.

Meanwhile, results by cause of death in Tables S13, S15, and S17 in Appendix S1 show that the RealAge score is as predictive as age for heart disease death (risk ratio for a unit change in age: 1.088, RealAge delta: 1.105), less predictive (but still significant) for cancers (age: 1.094, RealAge delta: 1.054), and more predictive of deaths from external causes (age: 0.992, RealAge delta: 1.090). Tables S14, S16, and S18 in Appendix S1 show substantively equivalent results with age in the base hazard. We note, in results not shown here, that age is predictive for deaths from external causes within age groups 25 to 44 and 65 to 84 (with coefficients 1.03 and 1.05), while coefficients for the RealAge delta does not vary from the full group (25 to 84) to the age subgroups.

### Estimates for Users with at Least Two Years of Follow-up and No Reported Prior Diagnosis

Table S19 in Appendix S1 shows that results do not change when we restrict our analysis to users who registered on the site at least 2 years prior to death. This finding provides some evidence that people who increase their search for health information immediately following a diagnosis are not driving the relationships we observe.

Age and RealAge maintain approximately equal accuracy for deaths from heart disease among users with no reported history of heart attack or stroke (or reported health interest in these topics). Also, the discrepancy between age and RealAge for cancer deaths among those with no prior interest or reported cancer diagnosis is constant between the cancer diagnosis and no cancer diagnosis models. As before, the RealAge score is predictive of deaths from external causes for users with no prior interest or reported depression diagnosis, while age is not predictive for these deaths. These results provide preliminary evidence that the overall comparisons of age and Realage are not substantially confounded by illness-only information-seeking behaviors.

**Figure 2:**
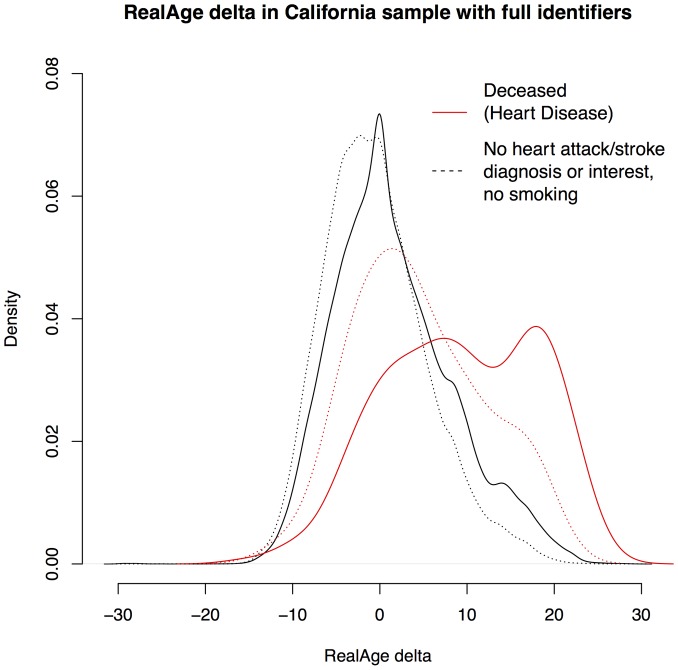
Comparison of RealAge score distributions for all deceased/surviving users and deceased/surviving “healthy” users - heart disease. The red lines indicate that the users are deceased and black lines indicate that the users were not identified as deceased. The dotted lines indicate that the group excluded users with a history of heart attack, stroke (or interest in either of heart attack or stroke topics), or smoking.

Tables S20, S21, and S22 show the results with smokers excluded (the external causes table is no depression only). The heart disease and external cause analyses are not substantially altered by the restrictions. However, the RealAge score does not predict cancer deaths among this specific “healthy”, never-smoked group of users. Figures 2, S12 (a duplicate of Figure 2 for format consistency), S13, and S14 in Appendix S1 compare the distribution of the RealAge delta among deceased/surviving users with no health restrictions to deceased/surviving users with the health and interest restrictions in this sensitivity analysis.

## Discussion

In this online context that relies on self-reported medical information, the RealAge score outperforms the Framingham ATP-III model. This is perhaps in part because additional inputs make the RealAge model less reliant on the potentially inaccurate blood pressure and cholesterol level reports. This result suggests that factors which are relatively simple and unlikely to be consistently misreported may lead to improved health assessments in online ecologies.

The validation of the RealAge score in its user population is appropriate because the score has been assigned to this group. However, our results are not necessarily generalizable beyond the RealAge user base. The RealAge user population is not representative of the US population at large, though it may be relatively representative of the part of the population that is mostly white, female, college-educated, older, and seeking health information (see Table 1 for summary statistics).

There is some indication that users fill out ‘what-if’ conditions and health statuses (such as different weights and cancer histories). This behavior will reduce the efficiency of our models and very likely attenuate the coefficient estimates on condition variables. Estimates of the effects of specific conditions on health outcomes would likely be better evaluated through verified diagnoses.

Further, many users may incorrectly self-diagnose. However, use of self-reported data to evaluate the RealAge score is appropriate because the users obtain this health metric from their self-reports. The metric is only useful to the extent that it on average predicts mortality in the presence of some user error.

There is some evidence that the use of RealAge leads to lower reported waist circumference [25] and, on average, we observe weight loss (a few pounds for each new update that does not appear to be a ‘what-if’ weight report) among RealAge users who take the test more than once. For our purposes here, this means that the RealAge scores of surviving users may decrease (slightly) over time, and this would lead to lower RealAge scores for surviving users overall.

We note that RealAge recommends behavior changes and medications to RealAge survey takers, and that these recommendations are personalized according to the RealAge score results. For example, many users at a higher risk of heart disease will be recommended a low daily dose of aspirin and cholesterol lowering medications. Whether these RealAge score-related recommendations affect the course of illness is an avenue of future study.

Finally, we cannot yet fully address the possibility that terminally ill patients who use the site are more likely to have high RealAge scores that do not reflect their general health prior to their illness. However, our analyses restricting time to death and cause of death suggest that the results are insensitive to this confounder.

## Supporting Information

Appendix S1 **Figure S1** Comparison of death rates in RealAge sample to US death rates. **Figure S2** RealAge delta in California sample with full identifiers. **Figure S3** RealAge delta in RealAge population. **Figure S4** RealAge delta in California sample with full identifiers - age 25 to 44. **Figure S5** RealAge delta in California sample with full identifiers - age 45 to 64. **Figure S6** RealAge delta in California sample with full identifiers - age 65 to 84. **Figure S7** Calibration plot - Age. **Figure S8** Calibration plot - RealAge. **Figure S9** Calibration plot - Framingham. **Figure S10** Calibration plot - Framingham (unscaled age). **Figure S11** Calibration plot - Framingham (unscaled age, indicators only). **Figure S12** Comparison of RealAge score distributions for all deceased/surviving users and deceased/surviving “healthy” users (heart disease). **Figure S13** Comparison of RealAge score distributions for all deceased/surviving users and deceased/surviving “healthy” users (cancer). **Figure S14** Comparison of RealAge score distributions for all deceased/surviving users and deceased/surviving “healthy” users (external causes). **Table S1** 25 to 44 - causes of death. **Table S2** 45 to 64 - causes of death. **Table S3** 65 to 84 - causes of death. **Table S4** Log-Likelihood for Table 4 Comparison of Age and RealAge. **Table S5** Framingham Hard Coronary Heart Disease 10-Year Risk Model results–unscaled age. **Table S6** Framingham Hard Coronary Heart Disease 10-Year Risk Model results–indicators only, unscaled age. **Table S7** 25 to 44 - model results. **Table S8** 25 to 44 - model results (age base). **Table S9** 45 to 64 - model results. **Table S10** 45 to 64 - model results (age base). **Table S11** 65 to 84 - model results. **Table S12** 65 to 84 - model results (age base). **Table S13** All users - Death from Heart Disease. **Table S14** Death from Heart Disease (age in base hazard). **Table S15** All users - Death from Cancer. **Table S16** Death from Cancer (age in base hazard). **Table S17** All users - Death from External Cause. **Table S18** Death from External Cause (age in base hazard). **Table S19** All users - at least two years follow-up. **Table S20** All users - Death from Heart Disease, no Report Diagnosis or Interest for Heart Attack or Stroke, no Smoking. **Table S21** All users - Death from Cancer, no Reported Diagnosis or Interest for Cancer, no Smoking. **Table S22** All users - Death from External Cause, no Reported Diagnosis or Interest for Depression.
(PDF)

## References

[1] Harris Interactive, Inc (2010) Harris Poll: Cyberchondriacs” on the rise? Those who go online for healthcare information continues to increase.

[2] Pew Internet and American Life Project (2013) Online Health 2013. Pew Research Center's Internet & American Life Project.

[3] Morahan-Martin JM (2004) How internet users find, evaluate, and use online health information: a cross-cultural review. CyberPsychology & Behavior 7: 497–510. 15667044 10.1089/cpb.2004.7.497

[4] Hesse BW, Nelson DE, Kreps GL, Croyle RT, Arora NK, et al (2005) Trust and sources of health information: the impact of the Internet and its implications for health care providers: findings from the first Health Information National Trends Survey. Archives of Internal Medicine 165: 2618. 16344419 10.1001/archinte.165.22.2618

[5] Sillence E, Briggs P, Harris PR, Fishwick L (2007) How do patients evaluate and make use of online health information? Social Science & Medicine 64: 1853–1862. 17328998 10.1016/j.socscimed.2007.01.012

[6] Zulman DM, Kirch M, Zheng K, An LC (2011) Trust in the Internet as a Health Resource Among Older Adults: Analysis of Data from a Nationally Representative Survey. Journal of Medical Internet Research 13: e19. 21324832 10.2196/jmir.1552 PMC3221340

[7] Krebs P, Prochaska JO, Rossi JS (2010) A meta-analysis of computer-tailored interventions for health behavior change. Preventive Medicine 51: 214–221. 20558196 10.1016/j.ypmed.2010.06.004 PMC2939185

[8] Winker MA, Flanagin A, Chi-Lum B, White J, Andrews K, et al (2000) Guidelines for medical and health information sites on the internet. The Journal of the American Medical Association 283: 1600–1606. 10735398 10.1001/jama.283.12.1600

[9] Eysenbach G, Powell J, Kuss O, Sa ER (2002) Empirical Studies Assessing the Quality of Health Information for Consumerson the World Wide Web. The Journal of the American Medical Asso-ciation 287: 2691–2700. 10.1001/jama.287.20.2691 12020305

[10] Diaz JA, Griffith RA, Ng JJ, Reinert SE, Friedmann PD, et al (2002) Patients' use of the Internet for medical information. Journal of General Internal Medicine 17: 180–185. 11929503 10.1046/j.1525-1497.2002.10603.x PMC1495021

[11] Schwartz KL, Roe T, Northrup J, Meza J, Seifeldin R, et al (2006) Family medicine patients' use of the Internet for health information: a MetroNet study. The Journal of the American Board of Family Medicine 19: 39–45. 16492004 10.3122/jabfm.19.1.39

[12] World Health Organization (1990) International Statistical Classification of Diseases and Health Related Problems, Tenth Edition. Geneva: World Health Organization.

[13] Cox DR (1972) Regression models and life-tables. Journal of the Royal Statistical Society Series B (Methodological): 187–220.

[14] Allison PD (1984) Event history analysis: Regression for longitudinal event data. Sage Publications, Incorporated.

[15] Steyerberg EW, Vickers AJ, Cook NR, Gerds T, Gonen M, et al (2010) Assessing the Performance of Prediction Models. Epidemiology 21: 128–138. 20010215 10.1097/EDE.0b013e3181c30fb2 PMC3575184

[16] Harrell FE (2001) Regression modeling strategies: with applications to linear models, logistic regression, and survival analysis. New York: Springer.

[17] Pencina MJ, D' Agostino RB, Vasan RS (2008) Evaluating the added predictive ability of a new marker: From area under the ROC curve to reclassification and beyond. Statistics in Medicine 27: 157–172. 17569110 10.1002/sim.2929

[18] Cook NR, Paynter NP, Eaton CB, Manson JE, Martin LW, et al (2012) Comparison of the Fram-ingham and Reynolds Risk Scores for Global Cardiovascular Risk Prediction in the Multiethnic Women's Health Initiative. Circulation 125: 1748–1756. 22399535 10.1161/CIRCULATIONAHA.111.075929 PMC3324658

[19] Executive Summary of the Third Report of the National Cholesterol Education Program (NCEP) Expert Panel on Detection, Evaluation, and Treatment of High Blood Cholesterol in Adults (Adult Treatment Panel III). The Journal of the American Medical Association 285: 2486–2497. 10.1001/jama.285.19.2486 11368702

[20] Mayer DK, Terrin NC, Kreps GL, Menon U, McCance K, et al (2007) Cancer survivors information seeking behaviors: A comparison of survivors who do and do not seek information about cancer. Patient Education and Counseling 65: 342–350. 17029864 10.1016/j.pec.2006.08.015 PMC5693234

[21] Hesse BW, Arora NK, Burke Beckjord E, Finney Rutten LJ (2008) Information support for cancer survivors. Cancer 112: 2529–2540. 18428201 10.1002/cncr.23445

[22] Weaver JB III, Mays D, Weaver SS, Hopkins GL, Eroğlu D, et al (2010) Health Information–Seeking Behaviors, Health Indicators, and Health Risks. American Journal of Public Health 100: 1520–1525. 20558794 10.2105/AJPH.2009.180521 PMC2901281

[23] Kom EL, Graubard BI, Midthune D (1997) Time-to-Event Analysis of Longitudinal Follow-up of a Survey: Choice of the Time-scale. American Journal of Epidemiology 145: 72–80. 8982025 10.1093/oxfordjournals.aje.a009034

[24] Thiébaut ACM, Bénichou J (2004) Choice of time-scale in Cox's model analysis of epidemiologic cohort data: a simulation study. Statistics in Medicine 23: 3803–3820. 15580597 10.1002/sim.2098

[25] Hughes SL, Seymour RB, Campbell RT, Shaw JW, Fabiyi C, et al (2011) Comparison of Two Health-Promotion Programs for Older Workers. American Journal of Public Health 101: 883–890. 21421955 10.2105/AJPH.2010.300082 PMC3076396

