# Supplementary material for: Prediction of Mortality Using On-Line, Self-Reported Health Data: Empirical Test of the Realage Score
Source: PLoS One. 2014 Jan 17;9(1):e86385. doi: 10.1371/journal.pone.0086385 (PMC3895041; doi:10.1371/journal.pone.0086385)
Supplement: Appendix S1 — Figure S1 Comparison of death rates in RealAge sample to US death rates. Figure S2 RealAge delta in California sample with full identifiers. Figure S3 RealAge delta in RealAge population. Figure S4 RealAge delta in California sample with full identifiers - age 25 to 44. Figure S5 RealAge delta in California sample with full identifiers - age 45 to 64. Figure S6 RealAge delta in California sample with full identifiers - age 65 to 84. Figure S7 Calibration plot - Age. Figure S8 Calibration plot - RealAge. Figure S9 Calibration plot - Framingham. Figure S10 Calibration plot - Framingham (unscaled age). Figure S11 Calibration plot - Framingham (unscaled age, indicators only). Figure S12 Comparison of RealAge score distributions for all deceased/surviving users and deceased/surviving “healthy” users (heart disease). Figure S13 Comparison of RealAge score distributions for all deceased/surviving users and deceased/surviving “healthy” users (cancer). Figure S14 Comparison of RealAge score distributions for all deceased/surviving users and deceased/surviving “healthy” users (external causes). Table S1 25 to 44 - causes of death. Table S2 45 to 64 - causes of death. Table S3 65 to 84 - causes of death. Table S4 Log-Likelihood for Table 4 Comparison of Age and RealAge. Table S5 Framingham Hard Coronary Heart Disease 10-Year Risk Model results–unscaled age. Table S6 Framingham Hard Coronary Heart Disease 10-Year Risk Model results–indicators only, unscaled age. Table S7 25 to 44 - model results. Table S8 25 to 44 - model results (age base). Table S9 45 to 64 - model results. Table S10 45 to 64 - model results (age base). Table S11 65 to 84 - model results. Table S12 65 to 84 - model results (age base). Table S13 All users - Death from Heart Disease. Table S14 Death from Heart Disease (age in base hazard). Table S15 All users - Death from Cancer. Table S16 Death from Cancer (age in base hazard). Table S17 All users - Death from External Cause. Table S18 Death from External Caus [file pone.0086385.s001.pdf]

# Appendix S1

## List of Figures


S1 *Comparison of death rates in RealAge sample to US death rates.* . . . 1
S2 *RealAge delta in California sample with full identifiers* . . . 2
S3 *RealAge delta in RealAge population* . . . 3
S4 *RealAge delta in California sample with full identifiers - age 25 to 44* . . . 5
S5 *RealAge delta in California sample with full identifiers - age 45 to 64* . . . 7
S6 *RealAge delta in California sample with full identifiers - age 65 to 84* . . . 9
S7 *Calibration plot - Age.* . . . 12
S8 *Calibration plot - RealAge.* . . . 13
S9 *Calibration plot - Framingham.* . . . 14
S10 *Calibration plot - Framingham (unscaled age)* . . . 15
S11 *Calibration plot - Framingham (unscaled age, indicators only)* . . . 16
S12 *Comparison of RealAge score distributions for all deceased/surviving users and deceased/surviving "healthy" users (heart disease).* . . . 21
S13 *Comparison of RealAge score distributions for all deceased/surviving users and deceased/surviving "healthy" users (cancer).* . . . 23
S14 *Comparison of RealAge score distributions for all deceased/surviving users and deceased/surviving "healthy" users (external causes).* . . . 25


## List of Tables


S1 25 to 44 - causes of death . . . 4
S2 45 to 64 - causes of death . . . 6
S3 65 to 84 - causes of death . . . 8
S4 Log-Likelihood for Table 4 Comparison of Age and RealAge . . . 10
S5 Framingham Hard Coronary Heart Disease 10-Year Risk Model results–unscaled age . . . 11
S6 Framingham Hard Coronary Heart Disease 10-Year Risk Model results–indicators only, unscaled age . 11
S7 25 to 44 - model results . . . 17
S8 25 to 44 - model results (age base) . . . 17
S9 45 to 64 - model results . . . 18
S10 45 to 64 - model results (age base) . . . 18
S11 65 to 84 - model results . . . 19
S12 65 to 84 - model results (age base) . . . 19
S13 All users - Death from Heart Disease . . . 20
S14 Death from Heart Disease (age in base hazard) . . . 20
S15 All users - Death from Cancer . . . 22
S16 Death from Cancer (age in base hazard) . . . 22
S17 All users - Death from External Cause . . . 24
S18 Death from External Cause (age in base hazard) . . . 24
S19 All users - at least two years follow-up . . . 26
S20 All users - Death from Heart Disease, no Report Diagnosis or Interest for Heart Attack or Stroke, no Smoking . . . 27
S21 All users - Death from Cancer, no Reported Diagnosis or Interest for Cancer, no Smoking . . . 27
S22 All users - Death from External Cause, no Reported Diagnosis or Interest for Depression . . . 28

## Comparison to US Death Rates

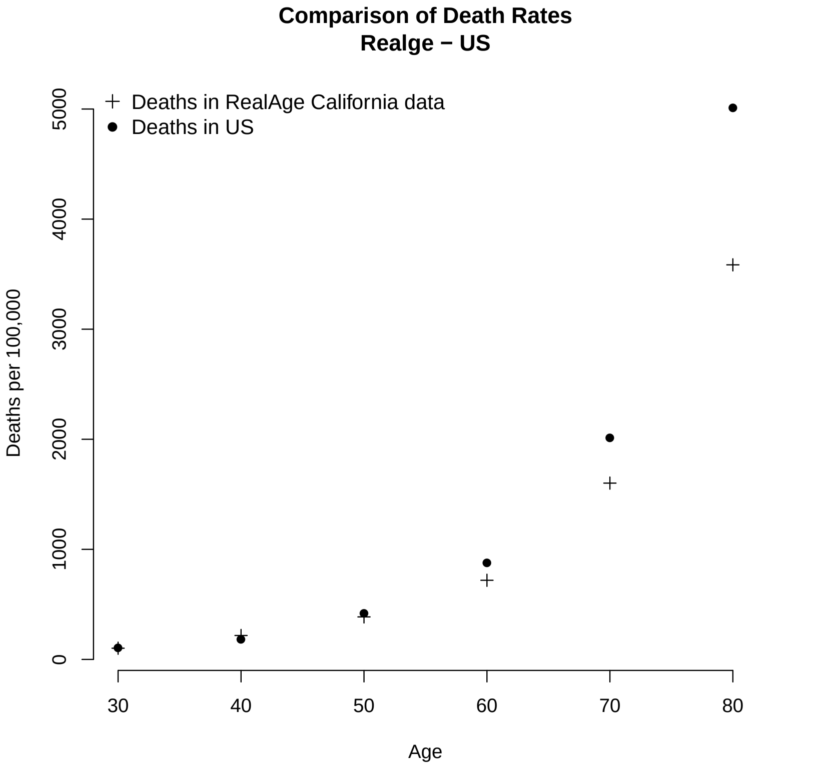


Figure S1: *Comparison of death rates in RealAge sample to US death rates.*

## Distribution of RealAge delta

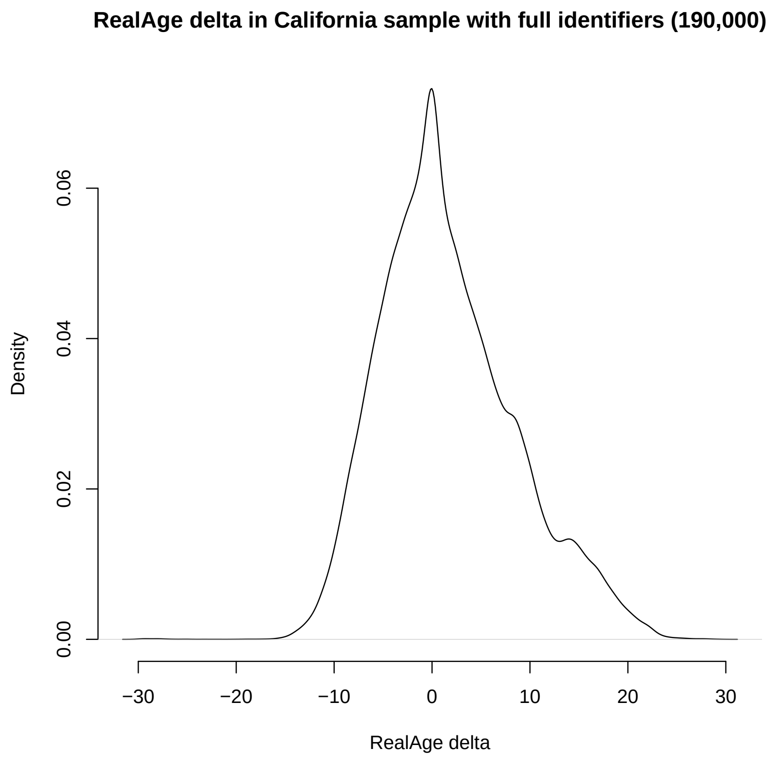


Figure S2: *RealAge delta in California sample with full identifiers*

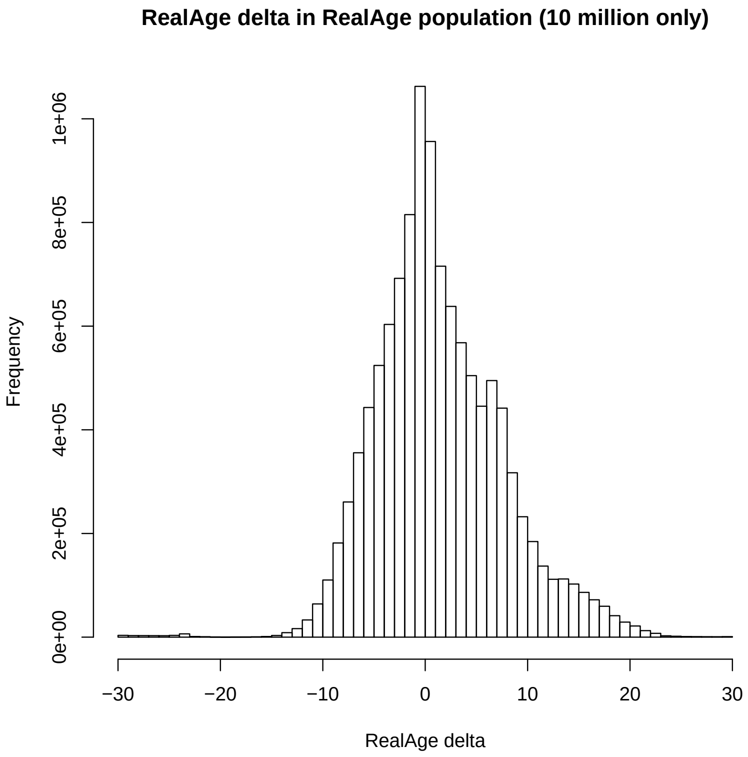


Figure S3: *RealAge delta in RealAge population*

## Causes of death and RealAge delta by age group

### 25 to 44

Table S1: 25 to 44 - causes of death

| Cause of Death | Count | Follow-Up Mean | Follow-Up Median |
|---|---|---|---|
| External Causes | 46 | 938.5 | 767 |
| Cancer | 21 | 1074 | 948 |
| Heart Disease | 16 | 1307.4 | 1041 |
| # of users in sample | 74,965 | | |
| # deceased in sample | 118 | | |

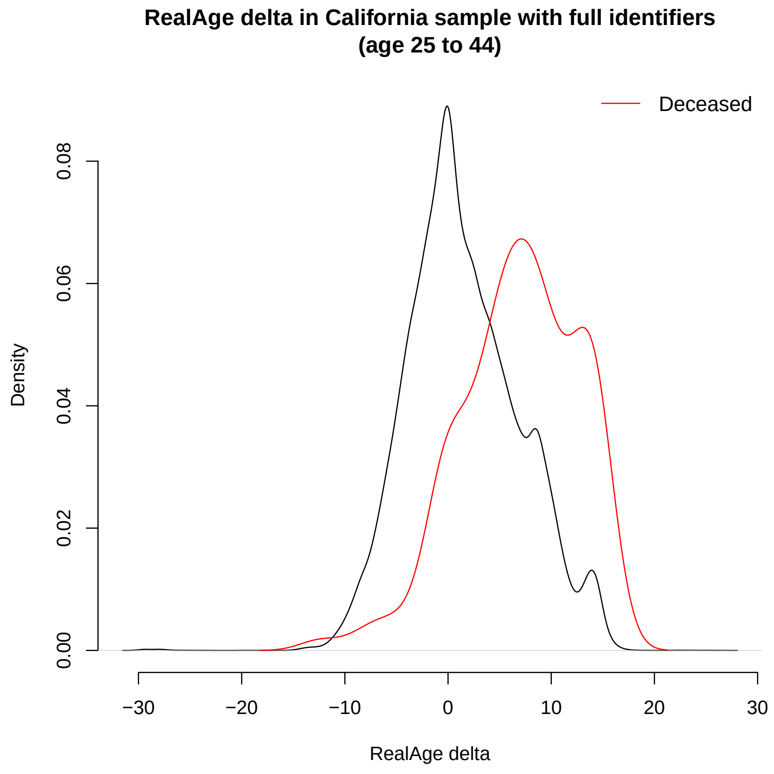


Figure S4: *RealAge delta in California sample with full identifiers - age 25 to 44*

**45 to 64**

Table S2: 45 to 64 - causes of death

| Cause of Death | Count | Follow-Up Mean | Follow-Up Median |
|---|---|---|---|
| Cancer | 161 | 1215.3 | 980 |
| Heart Disease | 115 | 970.4 | 779 |
| External Causes | 48 | 935.5 | 739 |
| Chronic Lower Respiratory | 29 | 993.2 | 887 |
| # of users in sample | 91,886 | | |
| # deceased in sample | 479 | | |

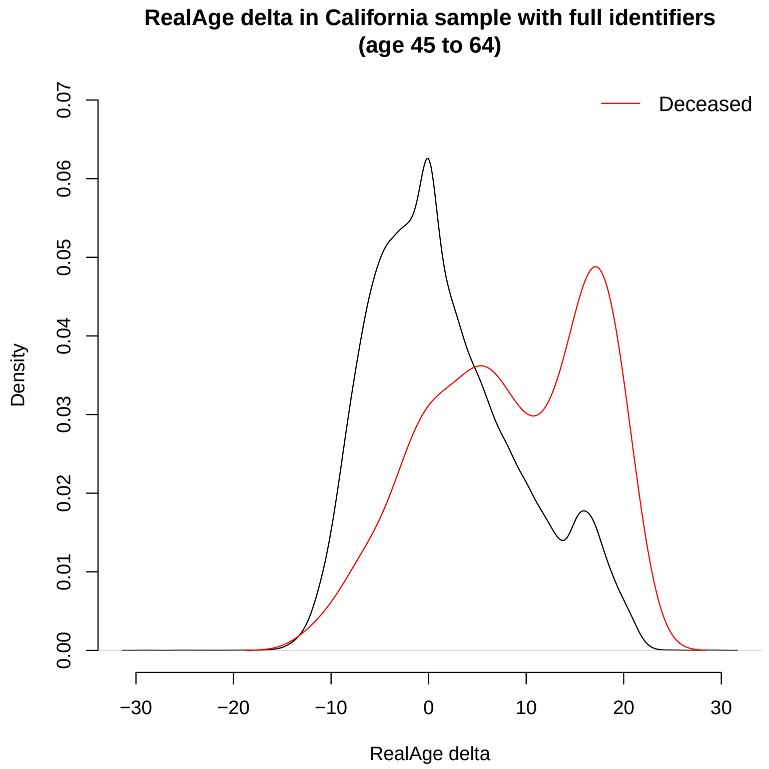


Figure S5: *RealAge delta in California sample with full identifiers - age 45 to 64*

**65 to 84**

Table S3: 65 to 84 - causes of death

| Cause of Death | Count | Follow-Up Mean | Follow-Up Median |
|---|---|---|---|
| Cancer | 157 | 1016.8 | 867 |
| Heart Disease | 121 | 1034 | 897 |
| Chronic Lower Respiratory | 29 | 1018.4 | 879 |
| Cerebrovascular Disease | 26 | 1120 | 1028.5 |
| # of users in sample | 21,847 | | |
| # deceased in sample | 449 | | |

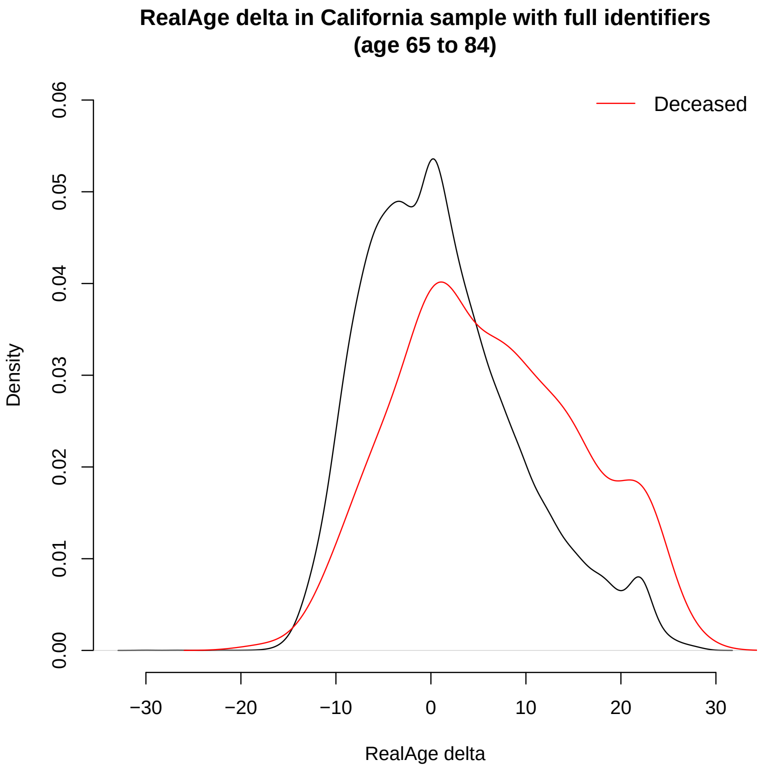


Figure S6: *RealAge delta in California sample with full identifiers - age 65 to 84*

## Log-Likelihood for Table 4 Comparison of Age and RealAge

Table S4: Log-Likelihood for Table 4 Comparison of Age and RealAge

| Model.1 | Log.Likelihood | |
|---|---|---|
| Age | -11176.1798, -10698.1112 | |
| | | |
| Model.2 | Log.Likelihood | chisq.df.p.value |
| RealAge | -10698.1112 -10416.2891 | 563.6442, 0, 0 |
| | | |
| Model.3 | Log.Likelihood | chisq.df.p.value |
| Age.and.RealAge.Delta | -10416.2891 -10414.563 | 3.4523, 1, 0.0632 |

## Framingham Risk Factor Estimates

Table S5: Framingham Hard Coronary Heart Disease 10-Year Risk Model results–unscaled age

| | HR | (2.5%, 97.5%) | p |
|---|---|---|---|
| Age | 1.073 | (1.054, 1.092) | 0.000 |
| Current Smoker | 4.218 | (2.858, 6.221) | 0.000 |
| Blood Pressure (points) | 1.266 | (1.062, 1.510) | 0.008 |
| Cholesterol (points) | 0.903 | (0.789, 1.033) | 0.136 |
| HDL Cholesterol (points) | 1.202 | (0.961, 1.504) | 0.108 |

Table S6: Framingham Hard Coronary Heart Disease 10-Year Risk Model results–indicators only, unscaled age

| | HR | (2.5%, 97.5%) | p |
|---|---|---|---|
| Age | 1.081 | (1.064, 1.097) | 0.000 |
| Current Smoker | 4.263 | (2.889, 6.290) | 0.000 |
| Blood Pressure | 1.567 | (1.085, 2.264) | 0.017 |
| Cholesterol | 0.732 | (0.494, 1.085) | 0.121 |

## Calibration plots

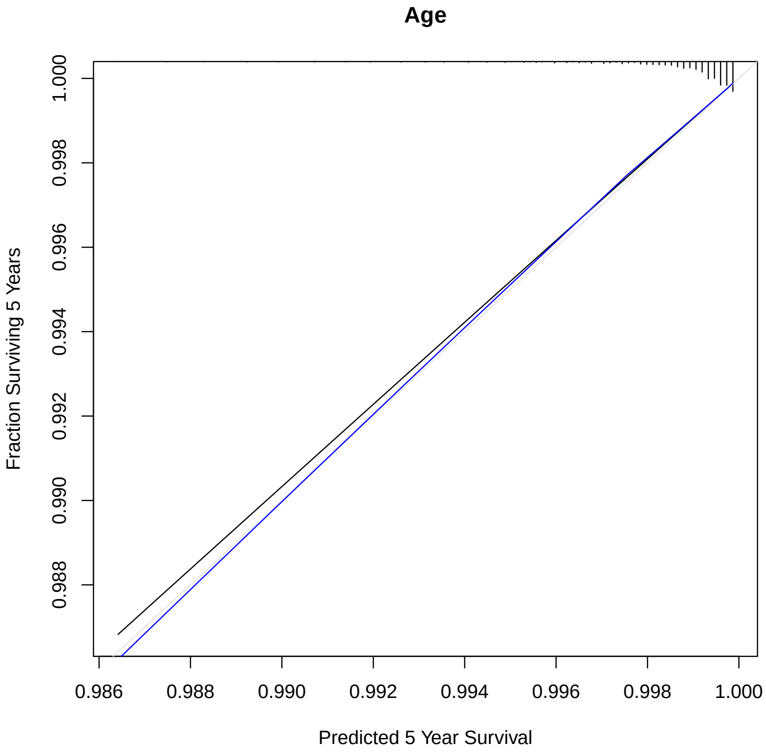


Figure S7: *Calibration plot - Age.*

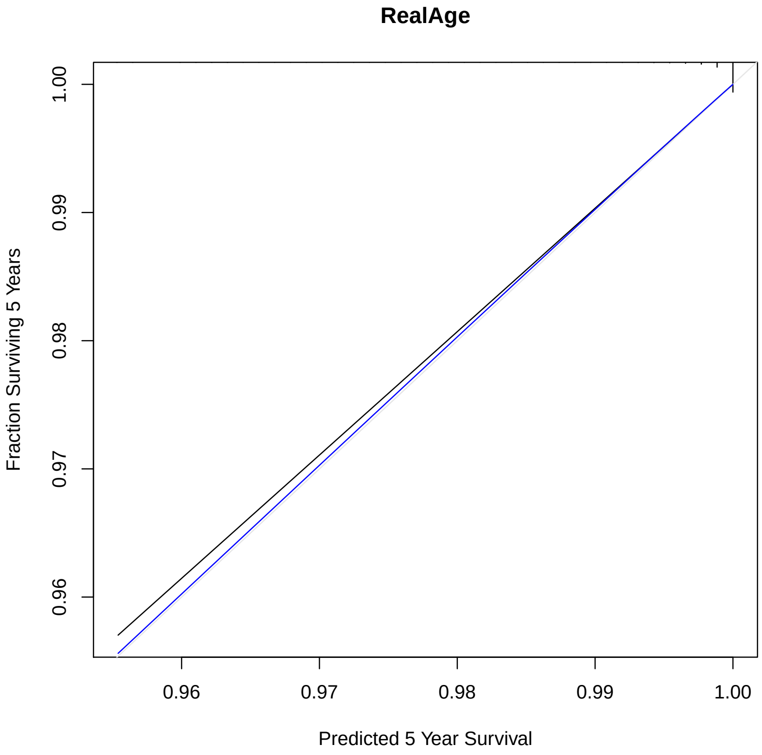


Figure S8: *Calibration plot - RealAge.*

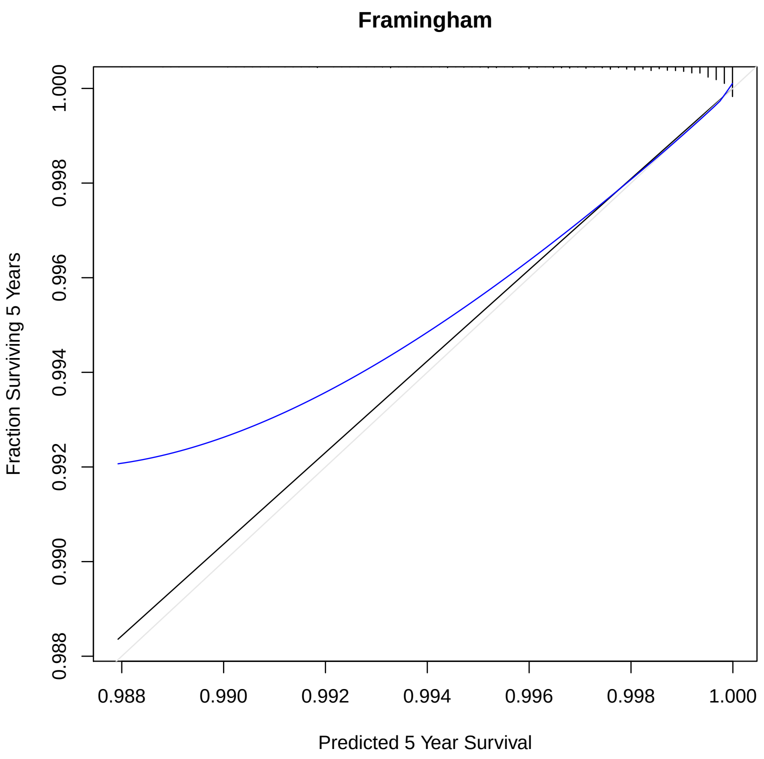


Figure S9: *Calibration plot - Framingham.*

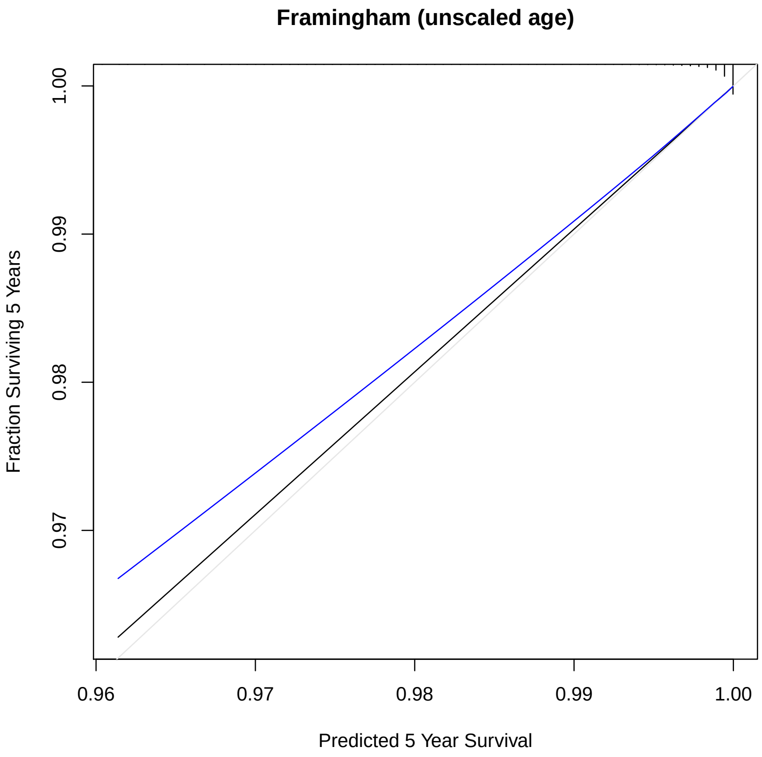


Figure S10: *Calibration plot - Framingham (unscaled age)*

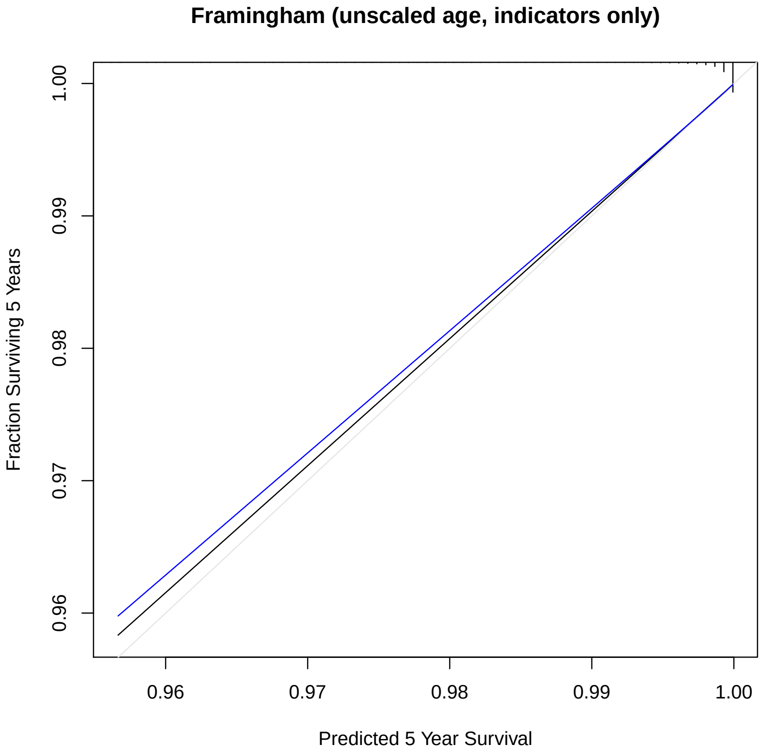


Figure S11: *Calibration plot - Framingham (unscaled age, indicators only)*

## Estimates by age group

### 25 to 44

Table S7: 25 to 44 - model results

| | HR | (2.5%, 97.5%) | p |
|---|---|---|---|
| Age | 1.054 | (1.021, 1.087) | 0.001 |

| | HR | (2.5%, 97.5%) | p |
|---|---|---|---|
| RealAge | 1.081 | (1.066, 1.095) | 0.000 |

| | HR | (2.5%, 97.5%) | p |
|---|---|---|---|
| Age | 1.049 | (1.017, 1.082) | 0.002 |
| RealAgeDelta | 1.087 | (1.073, 1.102) | 0.000 |

Table S8: 25 to 44 - model results (age base)

| | HR | (2.5%, 97.5%) | p |
|---|---|---|---|
| RealAgeDelta | 1.087 | (1.071, 1.103) | 0.000 |

**45 to 64**

Table S9: 45 to 64 - model results

| | HR | (2.5%, 97.5%) | p |
|---|---|---|---|
| Age | 1.068 | (1.050, 1.085) | 0.000 |

| | HR | (2.5%, 97.5%) | p |
|---|---|---|---|
| RealAge | 1.088 | (1.079, 1.096) | 0.000 |

| | HR | (2.5%, 97.5%) | p |
|---|---|---|---|
| Age | 1.064 | (1.047, 1.082) | 0.000 |
| RealAgeDelta | 1.094 | (1.085, 1.103) | 0.000 |

Table S10: 45 to 64 - model results (age base)

| | HR | (2.5%, 97.5%) | p |
|---|---|---|---|
| RealAgeDelta | 1.095 | (1.084, 1.105) | 0.000 |

**65 to 84**

Table S11: 65 to 84 - model results

| | HR | (2.5%, 97.5%) | p |
|---|---|---|---|
| Age | 1.081 | (1.064, 1.100) | 0.000 |

| | HR | (2.5%, 97.5%) | p |
|---|---|---|---|
| RealAge | 1.074 | (1.064, 1.083) | 0.000 |

| | HR | (2.5%, 97.5%) | p |
|---|---|---|---|
| Age | 1.086 | (1.067, 1.104) | 0.000 |
| RealAgeDelta | 1.070 | (1.059, 1.081) | 0.000 |

Table S12: 65 to 84 - model results (age base)

| | HR | (2.5%, 97.5%) | p |
|---|---|---|---|
| RealAgeDelta | 1.071 | (1.060, 1.082) | 0.000 |

## Heart Disease Estimates

Table S13: All users - Death from Heart Disease

| | HR | (2.5%, 97.5%) | p |
|---|---|---|---|
| Age | 1.088 | (1.077, 1.099) | 0.000 |

| | HR | (2.5%, 97.5%) | p |
|---|---|---|---|
| RealAge | 1.096 | (1.087, 1.105) | 0.000 |

| | HR | (2.5%, 97.5%) | p |
|---|---|---|---|
| Age | 1.088 | (1.077, 1.100) | 0.000 |
| RealAgeDelta | 1.105 | (1.092, 1.119) | 0.000 |

| | | | |
|---|---|---|---|
| # of users in sample | 187,904 | | |
| # deceased in sample | 252 | | |

Table S14: Death from Heart Disease (age in base hazard)

| | HR | (2.5%, 97.5%) | p |
|---|---|---|---|
| RealAgeDelta | 1.109 | (1.095, 1.123) | 0.000 |

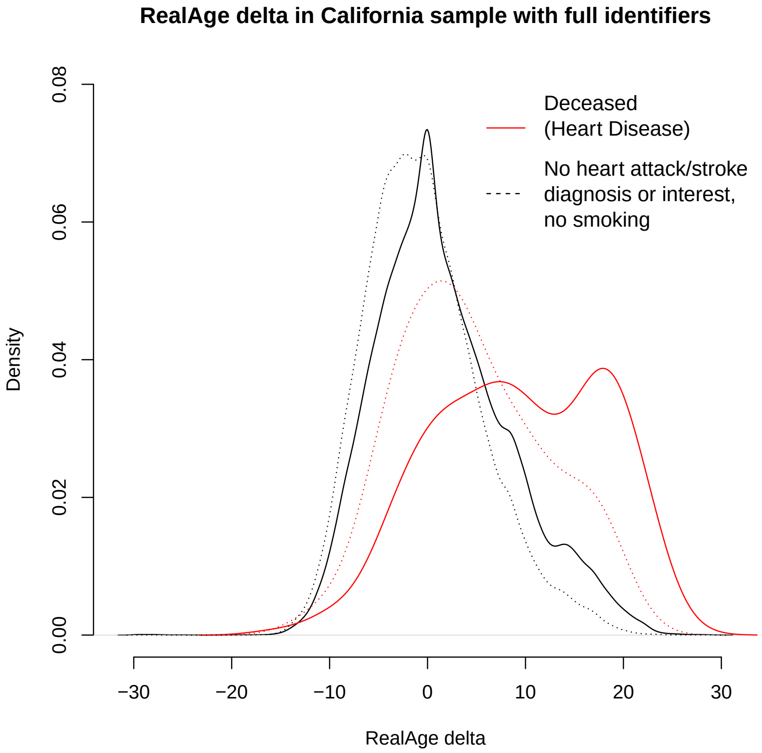


Figure S12: *Comparison of RealAge score distributions for all deceased/surviving users and deceased/surviving "healthy" users (heart disease).*

## Cancer Estimates

Table S15: All users - Death from Cancer

| | HR | (2.5%, 97.5%) | p |
|---|---|---|---|
| Age | 1.094 | (1.084, 1.103) | 0.000 |

| | HR | (2.5%, 97.5%) | p |
|---|---|---|---|
| RealAge | 1.079 | (1.071, 1.085) | 0.000 |

| | HR | (2.5%, 97.5%) | p |
|---|---|---|---|
| Age | 1.094 | (1.084, 1.103) | 0.000 |
| RealAgeDelta | 1.054 | (1.042, 1.067) | 0.000 |

| | | | |
|---|---|---|---|
| # of users in sample | 187,991 | | |
| # deceased in sample | 339 | | |

Table S16: Death from Cancer (age in base hazard)

| | HR | (2.5%, 97.5%) | p |
|---|---|---|---|
| RealAgeDelta | 1.051 | (1.039, 1.064) | 0.000 |

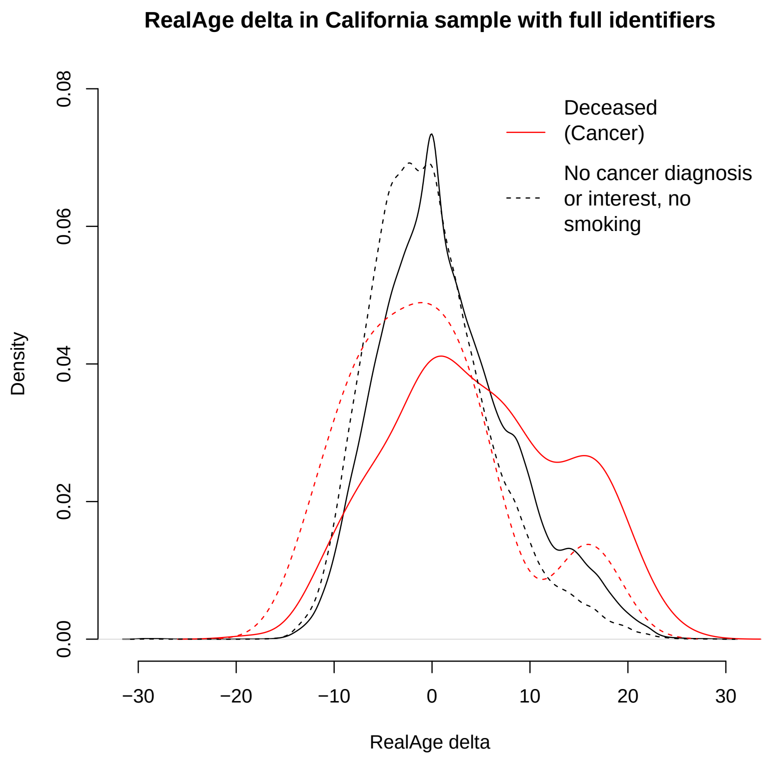


Figure S13: *Comparison of RealAge score distributions for all deceased/surviving users and deceased/surviving "healthy" users (cancer).*

## External Cause Estimates

Table S17: All users - Death from External Cause

| | HR | (2.5%, 97.5%) | p |
|---|---|---|---|
| Age | 0.996 | (0.980, 1.012) | 0.584 |

| | HR | (2.5%, 97.5%) | p |
|---|---|---|---|
| RealAge | 1.024 | (1.010, 1.038) | 0.000 |

| | HR | (2.5%, 97.5%) | p |
|---|---|---|---|
| Age | 0.992 | (0.976, 1.008) | 0.348 |
| RealAgeDelta | 1.090 | (1.070, 1.108) | 0.000 |

| | | | |
|---|---|---|---|
| # of users in sample | 187,741 | | |
| # deceased in sample | 89 | | |

Table S18: Death from External Cause (age in base hazard)

| | HR | (2.5%, 97.5%) | p |
|---|---|---|---|
| RealAgeDelta | 1.087 | (1.063, 1.111) | 0.000 |

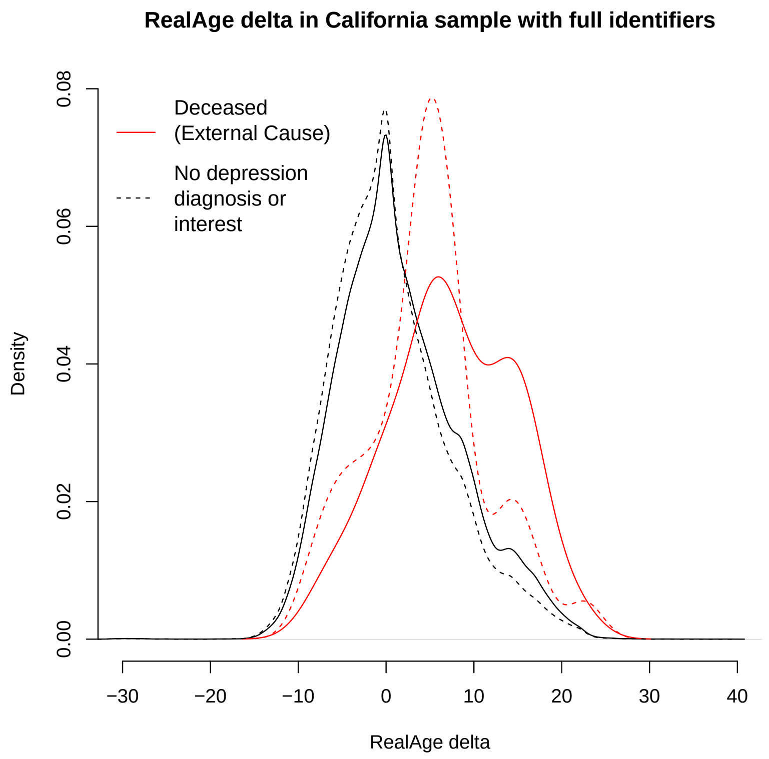


Figure S14: *Comparison of RealAge score distributions for all deceased/surviving users and deceased/surviving "healthy" users (external causes).*

## Estimates for users with at least two years of follow-up

Table S19: All users - at least two years follow-up

| | HR | (2.5%, 97.5%) | p |
|---|---|---|---|
| Age | 1.079 | (1.073, 1.085) | 0.000 |
| | HR | (2.5%, 97.5%) | p |
| RealAge | 1.080 | (1.075, 1.084) | 0.000 |
| | HR | (2.5%, 97.5%) | p |
| Age | 1.078 | (1.071, 1.085) | 0.000 |
| RealAgeDelta | 1.081 | (1.074, 1.090) | 0.000 |
| # of users in sample | 160,261 | | |
| # deceased in sample | 642 | | |

## Estimates for "healthy" users

Table S20: All users - Death from Heart Disease, no Report Diagnosis or Interest for Heart Attack or Stroke, no Smoking

| | HR | (2.5%, 97.5%) | p |
|---|---|---|---|
| Age | 1.071 | (1.046, 1.097) | 0.000 |
| | HR | (2.5%, 97.5%) | p |
| RealAge | 1.085 | (1.063, 1.107) | 0.000 |
| | HR | (2.5%, 97.5%) | p |
| Age | 1.076 | (1.050, 1.102) | 0.000 |
| RealAgeDelta | 1.103 | (1.070, 1.137) | 0.000 |
| # of users in sample | 84,190 | | |
| # deceased in sample | 40 | | |

Table S21: All users - Death from Cancer, no Reported Diagnosis or Interest for Cancer, no Smoking

| | HR | (2.5%, 97.5%) | p |
|---|---|---|---|
| Age | 1.108 | (1.083, 1.132) | 0.000 |
| | HR | (2.5%, 97.5%) | p |
| RealAge | 1.071 | (1.054, 1.088) | 0.000 |
| | HR | (2.5%, 97.5%) | p |
| Age | 1.108 | (1.083, 1.132) | 0.000 |
| RealAgeDelta | 1.004 | (0.968, 1.041) | 0.847 |
| # of users in sample | 71,271 | | |
| # deceased in sample | 52 | | |

Table S22: All users - Death from External Cause, no Reported Diagnosis or Interest for Depression

| | HR | (2.5%, 97.5%) | p |
|---|---|---|---|
| Age | 0.992 | (0.965, 1.020) | 0.594 |

| | HR | (2.5%, 97.5%) | p |
|---|---|---|---|
| RealAge | 1.010 | (0.985, 1.035) | 0.440 |

| | HR | (2.5%, 97.5%) | p |
|---|---|---|---|
| Age | 0.991 | (0.963, 1.019) | 0.509 |
| RealAgeDelta | 1.066 | (1.019, 1.115) | 0.005 |

| | |
|---|---|
| # of users in sample | 124,919 |
| # deceased in sample | 27 |
